# A Cyclic BMP-2 Peptide Upregulates BMP-2 Protein-Induced Cell Signaling in Myogenic Cells

**DOI:** 10.3390/polym13152549

**Published:** 2021-07-31

**Authors:** Vijaya Narasimha Gudivada, Chen-Ji Huang, Yueh-Hsia Luo, Guo-Chung Dong

**Affiliations:** 1 Institute of Biomedical Engineering and Nanomedicine, National Health Research Institutes, Miaoli 350, Taiwan; 044035@nhri.org.tw (V.N.G.); cjhuang@nhri.edu.tw (C.-J.H.); 2 Department of Life Sciences, National Central University, Taoyuan City 32001, Taiwan; yhluo@g.ncu.edu.tw

**Keywords:** bone morphogenetic protein (BMP-2), BMP-2 knuckle epitope, disulphide bridging, cyclic peptide

## Abstract

In the current study, we designed four cyclic peptide analogues by incorporating two cysteine residues in a BMP-2 linear knuckle epitope in such a way that the active region of the peptide could be either inside or outside the cyclic ring. Bone morphogenetic protein receptor BMPRII was immobilized on the chip surface, and the interaction of the linear and cyclic peptide analogues was studied using surface plasmon resonance (SPR). From the affinity data, the peptides with an active region inside the cyclic ring had a higher binding affinity in comparison to the other peptides. To confirm that our affinity data are in line in vitro, we studied the expression levels of RUNX2 (runt-related transcription factor) and conducted an osteogenic marker alkaline phosphatase (ALP) assay and staining. Based on the affinity data and the in vitro experiments, peptide P-05 could be a suitable candidate for osteogenesis, with higher binding affinity and increased RUNX2 and ALP expression in comparison to the linear peptides.

## 1. Introduction

Growth factors play a major role in the wound healing process by recruiting different cell types (chemotaxis) at the site of injury and also reducing the time for healing by enhancing the cell signaling [1,2]. There are many growth factors involved in the wound healing process, out of which there are more than 30 ligands of the TGF-β super family cloned and isolated, and only a few of them were characterized [3,4]. Bone morphogenetic proteins (BMPs) were originally identified in bone and cartilage; however, BMPs also have functions in other tissues, such as the teeth, kidneys, skin, hair, and muscles, and influence hematopoietic and neuronal development and maintain the iron metabolism and vascular homeostasis in vivo [5]. Among all of the BMPs, BMP-2 protein is approved by the United States Food and Drug Administration (US-FDA) in bone fracture healing, as well as in new bone formation, by promoting osteoprogenitor cells to osteogenesis [6,7,8]. In 2002, the (US-FDA) approved BMP-2 protein for the treatment of anterior lumbar tapered interbody fusion (ALIF) [9,10]. Bone grafts are used for the recovery of a fractured bone, which takes a longer time for recovery, whereas bone grafts combined with BMP-2 protein require less time [9]. There was extensive usage of rhBMP-2 protein bone grafts from 2002 to 2007, and many adverse cases of BMP-2 usage have been reported [9]. In 2008, the FDA issued an alert that the usage of BMP-2 protein in spinal fusion in certain cases can lead to swelling of the neck and throat tissue, resulting in compression of the airway and/or the neurological structures in the neck [11]. Sometimes, it can be life-threatening. The functionalization of biomaterials with full-length BMP-2 proteins is having the drawback of losing its activity. To overcome the shortcomings of BMP-2, a new approach utilizing small peptides containing a receptor-binding sequence of natural protein ligands [12,13] is being explored. On the other hand, delivering the peptides can be achieved by immobilizing them on biomaterials or delivering them through hydrogels where injectable hydrogels containing Col-II facilitate chondrogenic differentiation towards cartilage regeneration **[14]**.

BMP-2 protein is, structurally, a cysteine knot protein with two epitopes, i.e., wrist epitope binds to Bone morphogenetic protein receptor IA BMPRIA and knuckle epitope binds to Bone morphogenetic protein receptor-II (BMPRII). From the knuckle epitope region, a synthetic oligopeptide derived from BMP-2 protein (68–87) induces ectopic osteoinduction in vivo in rat calf muscles, which could be an alternative for BMP-2 protein [15,16,17]. In 2003, Saito et al. fragmented the BMP-2 protein from the knuckle epitope region into seven small peptides, and all of these peptides (i.e., P1, P2, P3, P4, P4sp, P5, and P6) were screened for alkaline phosphatase (ALP) activity [18]. From these results, it was confirmed that the P4 peptide shows ALP activity [18]. The BMP-2 peptide P4 was conjugated to alginate hydrogel, which shifted the fate of (MSCs) toward osteogenesis [19]. It has also been reported that usage of peptide P4 in combination with RGD, where RGD helps in the attachment of cells to cell surface receptors and BMP-2 peptide P4 helps in differentiation [13,20]. In this study, we propose to increase the osteogenic potential of peptide P4.

Currently, there are many strategies to improve the activity and stability of peptides, such as branching, cyclization, and stapled peptides. Of these, cyclization is one of the most efficient strategies for improving the activity and stability of the peptides. The cyclization of peptides can be achieved by various methods, such as lactam bridging, sulfur-bridging, or cyclization through organic linkers; furthermore, cyclization can be head-to-tail, side chain-to-N-terminal, or side chain-to-C-terminal [21]. Sulfur-bridging is one of the approaches followed by peptide chemists; it helps in enhancing activity, higher resistance to proteases and improving selectivity [22]. In recent work that employed the computer-aided design of three residues—namely, Serine (Ser) 88, Leucine (Leu) 90, and Tyrosine (Tyr) 91—from the C-terminal region of the knuckle epitope, the residues were found to be anchor residues to bind to the BMPRII receptor [23]. Herein, we report the cyclization of the BMP-2 peptide by introducing cysteine residues at different positions. We synthesized one linear and four analogue cyclic peptides. It is known that the BMP-2 knuckle epitope peptide binds to the BMPRII receptor, so we studied the interaction of the peptides with BMPRII using surface plasmon resonance (SPR). The BMPRII receptor was immobilized on the chip surface, and we compared the binding affinity. We further studied the osteogenic activity of the peptide analogues on the C2C12 cell line. C2C12 cells, upon treatment with BMP-2 protein, convert the myogenic pathway to the osteogenic pathway [24].

## 2. Materials and Methods

### 2.1. Synthesis of Peptide

All reagents were used without further purification unless otherwise stated. Fmoc-protected amino acids were purchased from Sigma-Aldrich (St. Louis, MI, USA), and rink amide MBHA resin was purchased from Novabiochem International (Wood Dale, IL, USA). N, N-diisopropylethyl amine (DIPEA) was purchased from Sigma-Aldrich (St. Louis, MI, USA). N, N-dimethylformamide (DMF) was purchased from Alfaaesar (Matsonford Road, Radnor, PA, USA). Trifluoroacetic acid (TFA) was purchased from Alfaaesar (Lancashire Lancs, UK). BMPRII receptor (R&D, MN, USA) and protein G (abcam, Cambridge, UK). Cyclization of the peptide was carried out by air oxidation in 10% DMSO (Alfaaesar, Lancashire Lancs, UK) at pH 7.0. Peptide synthesis scheme was represented in supplementary Figure S1. Purification of the cyclic peptide was conducted using RP-HPLC (Agilent 1200 Series, Agilent Technologies, Waldbronn, Germany), followed by lyophilization.

### 2.2. Use of SPR for Binding Analysis of BMPRII and BMP-2 Knuckle Epitope Peptides

SPR was performed using SensiQ discovery ((ICX Nomadics, Oklahoma City, OK, USA) SPR. For the affinity experiments, all the samples were dissolved in phosphate-buffered saline (PBS, pH 7.4) (UniRegion Bio-Tech, New Taipei City, Taiwan) containing 150 mM NaCl (St. Louis, MI, USA). The same buffer was also used as the running buffer. To immobilize the BMPRII, the active and reference flow cells of a COOH5 sensor chip (ICX Nomadics, Oklahoma City, OK, USA) carboxylic chip were activated with 1-ethyl-3-(3-(dimethylamino)-propyl) carbodiimide hydrochloride (EDC) and N-hydroxysuccinimide (NHS) (Cytiva, Marlborough, MA, USA) for 5 min. BMPRII protein diluted to 20 μg/mL with acetate buffer (pH4.5) (APOLO biochemical Inc., New York, NY, USA) was injected over the active cell for 3 min at 10 μL/min, resulting in an immobilization level of 2000 RU. Both the reference flow cell and active flow cell were blocked with ethanolamine (Bio Rad, Hercules, CA, USA), pH 8.5 for 6 min. The system was then switched to running buffer, and different concentrations of the peptide were injected for 120 s at 5 μL/min, with 5 min of dissociation. The binding data were analyzed using the BIA-evaluation software 3.0 (BIACORE, Uppasala, Sweden).

### 2.3. Cells and Cell Cultures

C2C12 cells (clone8, ATCC CRL-1772) cells were obtained from the Bio-Resource Collection and Research Center (Hsinchu, Taiwan), and were cultured in Dulbecco’s minimum essential medium (DMEM; Biological Industries, Kibbutz Beit-Haemek, Israel) containing 10% fetal bovine serum (FBS; Hyclone Laboratories, Logan, UT, USA) at 37 °C in 5% CO_2_.

### 2.4. Measurement of ALP Activity

A total of 3 × 10^4^ C2C12 cells were seeded on a 24-well plate in 10%FBS (Peak serum, Nicaragua, USA). After 24 h, the cells were treated with BMP-2 protein (2.5 nM) and BMP-2 protein with peptides 500 nM in 2% horse serum. The medium was changed after 3 days of treatment. Six days after treatment, the cells were washed with PBS, and then the cells were lysed in RIPA buffer (ab156034, abcam, Cambridge, UK), this solution was further centrifuged, and then the supernatant was collected, and the ALP assay procedure was followed, as given in Abcam (ab83369, abcam, Cambridge, UK). The absorbance of the sample was collected in a microplate reader at OD 405 (Thermo, Marsiling, Singapore). A standard curve is plotted, and from which the ALP activity was calculated using an equation. ALP assay was performed 3 times.

(1) $$ALp\;activity=\frac{B}{T*V}$$

B = amount of pNPP in sample well calculated from standard curve (µmol).
T = reaction time (minutes).
V = original sample volume added into the reaction well (mL).
D = sample dilution factor.

### 2.5. ALP Staining

A total of 3 × 10^4^ C2C12 cells were seeded on a 24-well plate in 10%FBS. After 24 h, the cells were treated with BMP-2 protein (R&D, MN, USA) and BMP-2 protein with peptides in 2% horse serum (Hyclone, Marlborough, USA). The medium was changed after 3 days of treatment. Six days after treatment, the cells were washed with PBS, the cells were fixed with formaldehyde (St. Louis, MI, USA), acetone (St. Louis, MI, USA), and citric acid (St. Louis, MI, USA) in the ratio of (1.5:3.25:1.25). After fixation, the cells were treated with an ALP-staining kit (Sigma-Aldrich, Spruce street, St. Louis, MO, USA) for 15 min. The reagents were washed away using milliQ water (Millipore, MA, USA), the plates were observed under the optical microscope for staining. ALP staining was performed 3 times.

### 2.6. Western Blotting

C2C12 (Mouse myoblasts) cells were incubated with BMP-2 peptides, BMP-2 protein (positive control) peptides with BMP-2 protein, and cells without any treatment were taken as a control for six days in a 3.5 cm Petri dish (Corning, Oneont, NY, USA) with cell number 3 × 10^5^ cells. The medium was changed every three days with BMP-2 protein and peptides. The cells were harvested, re-suspended in lysis buffer containing protease inhibitors (ab65621, abcam, Cambridge, UK), and sonicated to obtain cell lysate. For Western blot experiments, 20 micrograms of each treated and untreated cell lysates were resolved on SDS-PAGE. RUNX2 (runt-related transcription factor) was detected by the antibodies from Abcam (ab23981, abcam, Cambridge, UK). RUNX2 was performed 3 times.

## 3. Results

### 3.1. Design and Synthesis of BMP-2 Peptides

**Figure 1:**
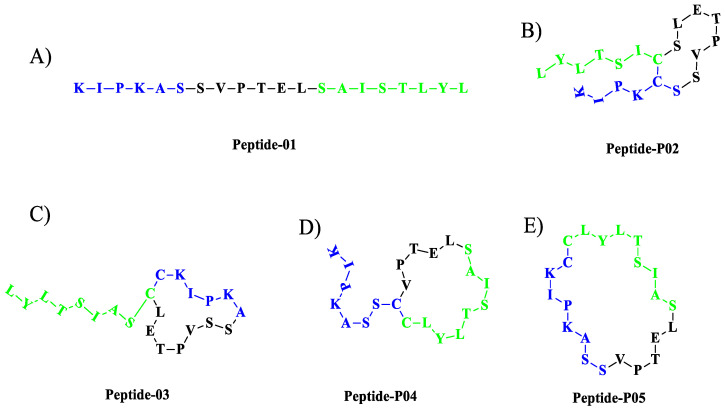
**Schematics of designed peptides:** (**A**) P-01: Linear knuckle epitope peptide; (**B**) P-02: Cyclic peptide, where the N-terminal and the C-terminal region’s (green) active sites were outside the cyclic structure; (**C**) P-03: The N-terminal was involved (blue) in the cyclic structure, while the C-terminal was free and outside of the cyclic structure; (**D**) P-04: The C-terminal (green) was involved in the cyclic structure, while the N-terminal was free and outside of the cyclic structure; (**E**) P-05: Both the N-terminal (blue) and the C-terminal (green) were involved in the cyclic structure.

Peptides P-01, P-02, P-03, P-04, and P-05 were designed from the BMPRII binding site of BMP-2. Peptide P01 is a linear peptide (73–92) region of BMP-2 protein, whereas P02, P03, P04, and P05 are cyclic peptides. Two cysteine residues were introduced into P02, P03, P04, and P05 to allow the possible stabilization of the peptide by forming a disulfide bond, as shown in Figure 1. In addition to the disulfide bond, the anchor residues Ser 88, Leu 91, and Tyr 92 in P-04 and P-05 were involved in the cyclic structure (C-terminal region), which helped the anchor residues to bind to BMPRII, and thus the structure became more rigid. The peptides designed were synthesized by solid-phase peptide synthesis (SPPS) and purified by high-performance liquid chromatography, and their ESI-MS results were observed as given in Table 1. The representative ESI-MS Figure S2 and chromatogram Figure S3 data were shown in supplementary information.

### 3.2. Interaction of the Peptides with BMPRII

**Figure 2:**
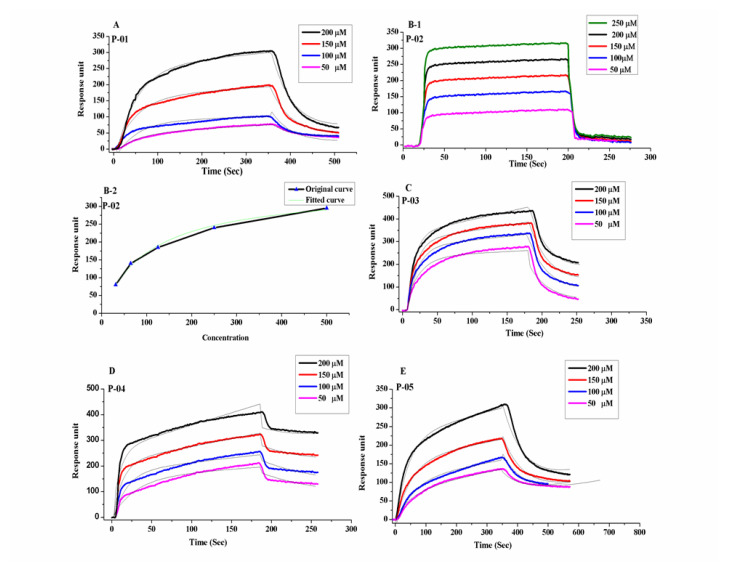
Surface plasmon resonance (SPR) sensograms of the peptides binding with immobilized BMPRII on the chip surface. (**A**) P-01; (**B-1**) P-02; (**B-2**) P-02 fitted curve; (**C**) P-03; (**D**) P-04; (**E**) P-05.

We first investigated the binding affinity of these five peptides for BMPRII using SPR. Each peptide was individually passed over a Sensiq CM5 chip pre-immobilized with BMPRII. As shown in Figure 2, P-01 had an equilibrium rate constant (KD) of 8.16 × 10^−2^ against BMPRII, whereas P-02, P-03, P-04, and P-05 had KD values of 8.06 × 10^−5^, 9.46 × 10^−5^, 2.31 × 10^−5^, and 1.29 × 10^−5^, respectively, which are also illustrated in Table 2. From the KD values obtained from SPR, it was observed that the P-05 cyclic BMP-2 peptide had a higher binding efficacy for the BMPRII receptor.

### 3.3. Osteogenic Differentiation of the C2C12 Cells

**Figure 3:**
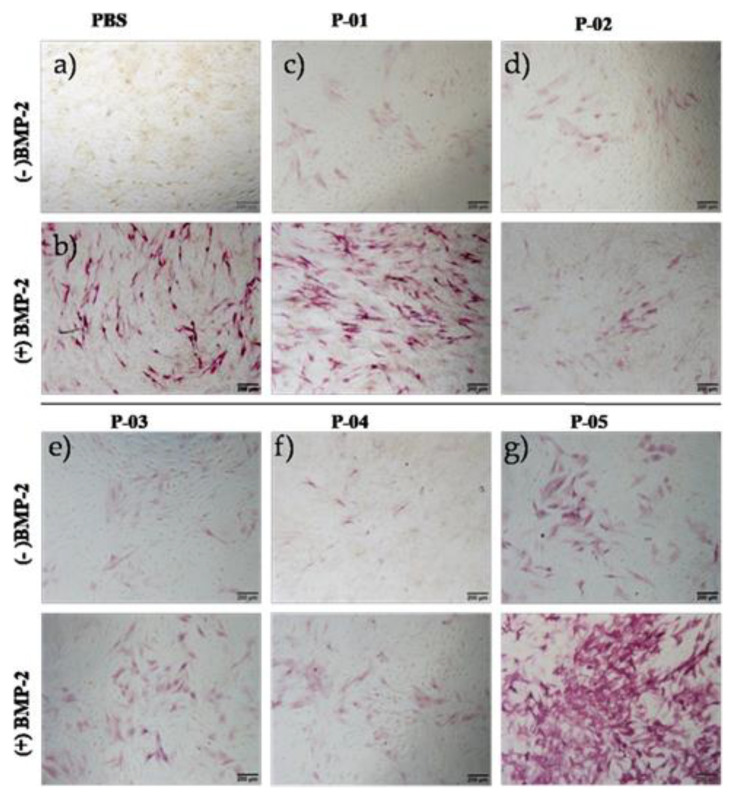
Alkaline phosphatase (ALP) staining in C2C12 cells (3 × 104 cells/well) cultured for six days in a 24-well plate, with peptides alone at a concentration of 500 nM. (- BMP) without BMP-2 protein; (+BMP-2) with BMP-2 protein; (**a**) Control with PBS; (**b**) BMP-2 protein 2.5 nM; (**c**) P-01; (**d**) P-02; (**e**) P-03; (**f**) P-04 (**g**) P-05.

We went on to access the activity of the peptides in vitro. We cultured C2C12 cells and peptides with medium for six days, and then the cells were fixed and stained for ALP. From the results, it is clear that for the peptides alone, the activity was significantly low Figure 3. In a previous publication [25], peptides were used in combination with the BMP-2 protein or in the presence of fetal bovine serum (FBS), as FBS contains a percentage of growth factors, so we performed ALP staining combined with a very low concentration of 2.5 nM of the BMP-2 protein. Peptides P-01 and P-05 had positive effects, whereas P-02, P-03, and P-04 had negative effects, which can clearly be observed in the ALP staining in Figure 3.

### 3.4. Effects of the Peptides and the Combination of Peptides in Cell Signaling

**Figure 4:**
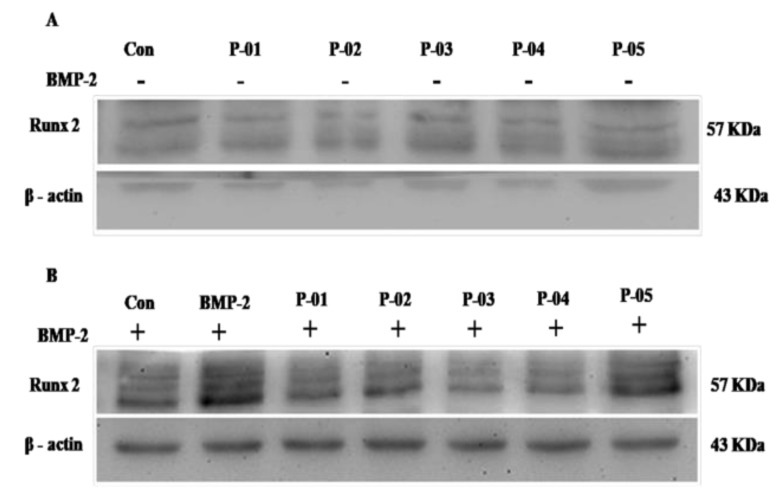
Western blot performed using the C2C12 cell line, with the blots treated using antiRUNX2 antibody. (**A**) Western blot of the RUNX2 expression of the control (without any treatment) and the different kinds of peptides (P-01 to P-05). (**B**) Western blot of the RUNX2 expression of the combination of the protein (2.5 nM) and peptides (500 nM) P-01 to P-05.

To confirm the results of the ALP staining, we further studied whether the cell signaling pathway related to osteogenesis is affected or not. We selected RUNX2 as a marker because RUNX2 is a common marker for both Smad and p38 signaling. We treated the cells with the BMP-2 peptides (Figure 4A) and a combination of the BMP-2 peptides with the BMP-2 protein (Figure 4B). From the results, it is clear that the expression of RUNX2 was not affected much by the BMP-2 peptide treatment. However, the RUNX2 expression was down-regulated in the combination treatment of the protein and peptides P-02, P-03, and P-04, whereas with peptide P-05, RUNX2 expression increased and with P-01, the expression was higher when compared to the other peptides.

### 3.5. Quantification of Alkaline Phosphatase (ALP)

**Figure 5:**
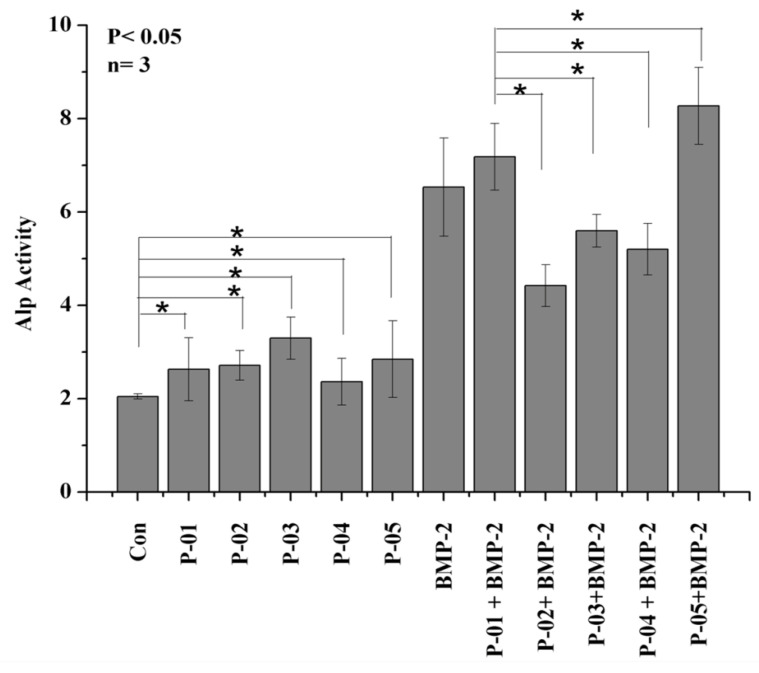
ALP activity in C2C12 cells (3 × 10^4^ cells/well) cultured for six days in 24-well plates with peptides alone at a concentration of 500 nM, as well as in combination with the BMP-2 protein (2.5 nM) and peptides at a concentration of 500 nM. The control group for the combination of the protein and peptides included peptide P-01 and the BMP-2 protein. * *p* < 0.05 compared to the control group (*n* = 3).

To quantify the differentiation of muscle cells (C2C12) to osteogenic cells, we estimated the amount of ALP released from the cells (Figure 5). Peptides alone from P-01 to P-05 slightly increased ALP when compared to the control. The peptides in combination with the protein provided different results: Peptide P-01 had a one-unit increase in ALP when compared to that of the BMP-2 protein. Meanwhile, for peptides P-02, P-03, and P-04, ALP was slightly decreased. Lastly, P-05 increased ALP by one and two units when compared with the combination of the BMP-2 protein and P-05 and the BMP-2 protein alone, respectively.

## 4. Discussion

The BMP-2 knuckle epitope peptide (BMP-2-KE) (73–92) has mostly been studied for the replacement of the BMP-2 protein [19,20,26,27]. Despite the extensive work on the BMP-2 peptide, the peptide’s active site and mechanism of action are still poorly understood. However, from previous studies, the binding site of BMP-2-KE has been shown to be in the C-terminal region, while the active site has been identified in three amino acids (i.e., Ser 88, Leu 90, and Tyr 91) in the binding region of the peptide [18,23]. The BMP-2 protein is a quaternary structure, by which amino acid Serine 88 can achieve possible orientation to interact with BMPRII at the regions of W85, S86, and H87 [28], in contrast to BMP2-KE, which is a linear peptide and does not have the proper orientation to interact with the BMPRII receptor at a specific site. So far, there have been no attempts made to study the active site information of the BMP-2-KE peptide. Moreover, as a linear peptide derived from Human acidic fibroblast growth factor (aFGF) converted to cyclic form, the cyclic peptide inhibitory dose (ID_50_ ≈ 30 μM) achieved a significant inhibitory effect against aFGF, binding to its receptor, in comparison to the linear peptide (ID50 > 1 mM) [29]. Therefore, we hypothesize that the cyclic peptide binds to BMPRII, by which it achieves a specific orientation. In another hypothesis, Activin receptor type II (ACVRII) and BMPRII are structurally similar and, hence, the BMP-2 peptide binds with either of them and makes the BMP-2 protein available for osteogenesis. Hence, it can initiate the oligomerization of receptors.

In the current study, we made an attempt to convert the BMP-2 knuckle epitope linear peptide to the cyclic peptide by incorporating sulfur residues to attain a specific orientation to interact with the receptor. P-05 (end-to-end cyclization), designed from the knuckle epitope peptide of the BMP-2 protein, increased the binding affinity more (with a KD value of 1.29 × 10^−5^ M) than did P-01 (8.16 × 10^−2^ M). According to a previous study, the BMP-2-KE peptide was used either in vitro, containing endogenous BMP-2, or in vivo, which was used in the presence of serum that contained growth factors such as TGF-β and BMP-2 [25]. Based on our findings, the BMP-2 peptide P-01- and P-05-treated C2C12 cells showed lower expression of RUNX2, whereas, in comparison to the combination of the BMP-2 peptide and protein, P-05 achieved higher expression of RUNX2 than P-01 with the protein.

The binding of P-05 to BMPRII is a preliminary event; furthermore, we studied the expression of the osteogenic marker RUNX2 in both the peptide-treated C2C12 cells, as well as in combination with the BMP-2 protein, which was relatively higher in comparison to the linear peptide in the BMP-2-induced C2C12 cell lines. Alkaline phosphatase is another characteristic marker for osteogenic cells, which has been shown to have higher ALP expression in C2C12 cells [17] when treated with P-05 (500 nM) in combination with the BMP-2 protein at a concentration of 2.5 nM. The BMP-2 knuckle epitope (KE) peptide was used in combination with the BMP-2 protein, as BMP-2 KE alone cannot induce osteogenesis.

Furthermore, we studied other peptides (i.e., P-02, P-03, and P-04). P-02 and P-03 were designed in such a way that the anchor residues were away from the cyclization. Peptide P-02 was designed to understand whether the free N- and C-terminals can enhance the peptide’s activity. Peptide P-03 was designed with a flanking C-terminal. The binding affinity of P-02 and P-03 was 9.46 × 10^−5^ M and 8.06 × 10^−5^ M, respectively, which was lower than that of P-04 and P-05. Interestingly, we observed that the RUNX2 expression of peptides P-02 and P-03, after ALP staining and quantification of ALP, was lower, even in the presence of the BMP-2 protein. This phenomenon may be due to peptides P-02 and P-03 having an antagonistic effect on the osteogenesis of the C2C12 cell line. The results of peptide P-03 showed that the flanking C-terminal region had a lower binding affinity and lower osteogenic potential.

The binding affinity of peptide P-04 (2.31 × 10^−5^ M) with BMPRII was higher in comparison to P-01, P-02, and P-03, and this higher affinity was due to the active sites being inside the cyclic ring, as well as the C-terminal region having a flanking N-terminal region. However, in comparison to P-05, the binding affinity of P-04 was slightly lower, and the in vitro activity of peptide P-04 was still lower than that of peptide P-05. Peptide P-04 also had a small cyclic ring in comparison to peptide P-05, and the size of this ring played an important role in the activity of the peptide.

In our current work, we have evaluated the activity of peptides using C2C12 cells as they are myogenic cells to study BMP signaling, so further evaluation of these peptides has to be performed in MSCs (Mesenchymal stem cells) to confirm the activity for tissue-engineering applications. We have also used some of the preliminary markers such as ALP and RUNX2 for studying the BMP-2 signaling pathway. Further, in our future work, we would like to extend our work to study the more mechanistic insights of cyclic BMP-2 peptides. However, peptides P-04 and P-05 had rigid structures in comparison to the other peptides; therefore, they had a higher binding affinity to the BMPRII receptor. Moreover, the ring size of the peptides affected their activity.

## 5. Conclusions

BMP-2 knuckle epitope peptide was extensively used in tissue-engineering applications; however, this was our first attempt to modify the peptide’s structure and increase its activity. From the results of affinity measurements and in vitro data, we have confirmed that peptide P-05 (end-to-end cyclization) has attained increased activity in comparison with linear BMP-2 peptides.

## Figures and Tables

**Table 1:** The molecular weight of the bone morphogenetic protein 2 (BMP-2) linear and cyclic peptides.

Compound Name	Structure and Molecular Weight (MW)	Observed Mass [M+2H]^2+^	Observed Mass [M+3H]^3+^
P-01	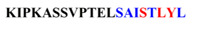	1059.62	706.74
P-02	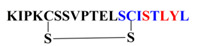	1090.8	726.53
P-03	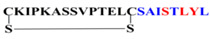	1161.61	774.74
P-04	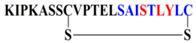	1161.61	774.74
P-05	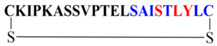	1161.61	774.74

**Table 2:** Association and dissociation rate constants of the peptides.

Compound Name	Association Rate Constant k_a_ (M^−1^s^−1^)	Dissociation Rate Constant k_d_ (s^−1^)	Equilibrium Rate Constant KD (M)
Linear peptide (P01)	2.7 × 10^−1^	2.21 × 10^−2^	8.16 × 10^−2^
N-and C-terminal free peptide (P-02)	Saturation binding curve	Saturation binding curve	8.06 × 10^−5^
N-terminal cyclic peptide (P-03)	3.52 × 10^2^	3.33 × 10^−2^	9.46 × 10^−5^
C-terminal cyclic peptide (P-04)	4.60 × 10^2^	1.06 × 10^−2^	2.31 × 10^−5^
N- to C-terminal cyclic peptide (P-05)	3.78 × 10^1^	4.87 × 10^−4^	1.29 × 10^−5^

## Data Availability

All data relevant to the study are included in the article.

## Supplementary Materials

The following are available online at https://www.mdpi.com/article/10.3390/polym13152549/s1, Figure S1: Solid-phase synthesis of peptide (P-05), Figure S2: ESI-MS spectrum of cyclic peptide P-05, Figure S3: HPLC profile of pure cyclic peptide P-05.
