# Supplementary material for: A Cyclic BMP-2 Peptide Upregulates BMP-2 Protein-Induced Cell Signaling in Myogenic Cells
Source: Polymers (Basel). 2021 Jul 31;13(15):2549. doi: 10.3390/polym13152549 (PMC8347162; doi:10.3390/polym13152549)
Supplement: Supplementary file 1 [file polymers-13-02549-s001.zip › polymers-1304902-SI.pdf]

Supplementary Materials:

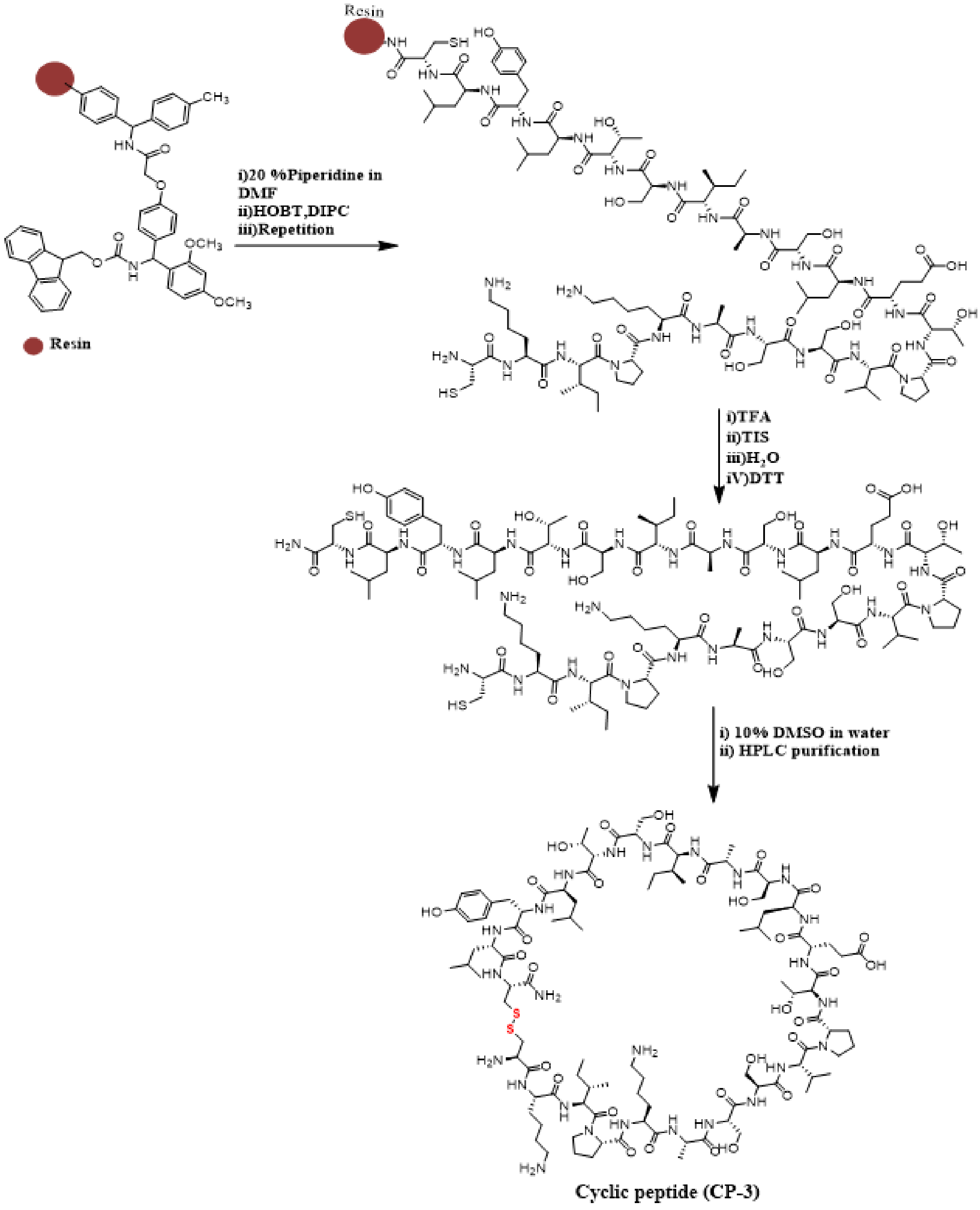

Figure S1. Solid-phase synthesis of peptide (P-05).

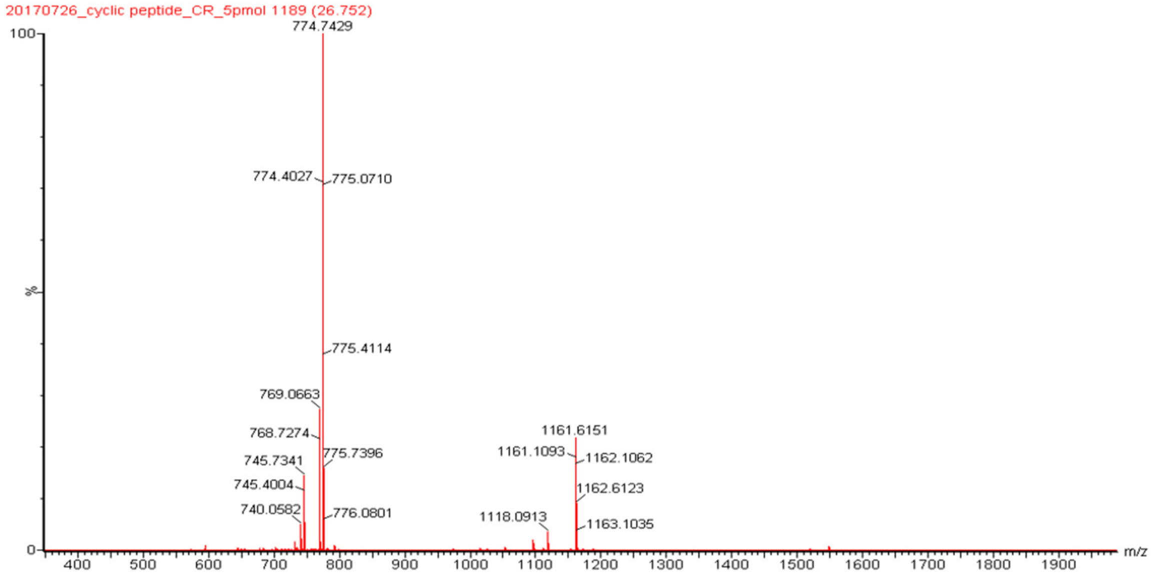

Figure S2. ESI-MS spectrum of cyclic peptide P-05.

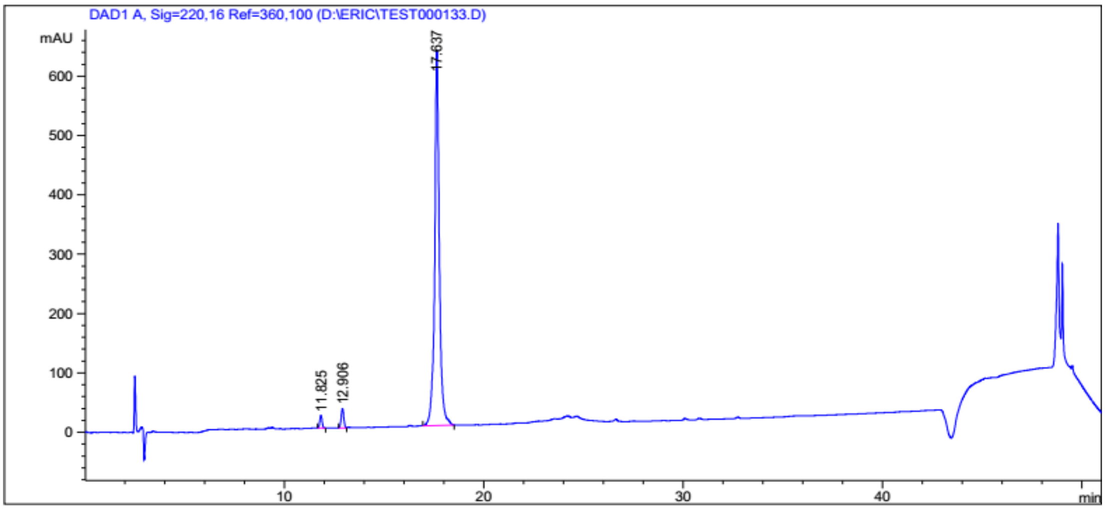

Figure S3. Hplc profile of pure cyclic peptide P-05.
